# The Prevention of Trade Accidents

**Published:** 1906-06-23

**Authors:** 


### The Prevention of Trade Accidents.

We have continual cause to be reminded of the truth of those sayings which have been described as the products of "the wit of one and the wisdom of many." The accuracy of such a saying as "familiarity breeds contempt" may be brought under our notice in many ways, but possibly it may have fallen to our lot to see it more than once illustrated in a painful manner. A railway porter who has crossed the line many hundreds of times may for once have misjudged the speed of a train; or a cyclist, thoroughly at home in traffic, may not have taken into consideration the chance of skidding when twisting ahead of an omnibus. Not a day passes but numerous instances occur in which those, whom experience ought to protect, suffer for need of caution.

In these modern times, when so large a proportion of the working population is engaged in some form of manufacture carried on mainly by machinery, the risk of injury occasioned by want of sufficient attention to dangers arising from rapidly moving wheels and cranks, or other parts of a machine, is greatly increased. A recent article states that about 80,000 accidents happen yearly in our factories and workshops, and 1,000 of these are of such a serious character as to cause death. Familiarity with personal risk must account for many of these accidents, yet it would be unreasonable to consider them all to be due to culpable carelessness. Disregard of danger is no doubt frequently an indication of absorption in the work of the moment, and may consequently be rather an indication of a praiseworthy quality than the reverse. However that may be, many injuries are apparently received as a result of some trifling circumstance that could scarcely have been avoided, such as the slipping of an engineer on the oily floor of the engine-room which brings him within striking distance of the machinery. Other mishaps not so purely to be attributed to unavoidable accidents are occasioned by a loose sleeve catching in a rapidly revolving shaft, or by stooping to pick up some dropped article close to a machine in motion. But in whatever way an accident may arise, whether indirectly as the result of energetic industry, or directly to carelessness, it is necessary that all possible means of prevention of such accidents should be adopted.

It is interesting to learn that endeavours are now made to protect those working in connection with machines from injury. The risk to an engineer of slipping in the engine-room and receiving a blow from the large moving beam can be obviated by fencing the beam on all sides with a foot-plate and double rails. Fly-wheels are protected with wire guards; and moving shafts, in positions where they could give rise to injury, are also surrounded by boarding or fencing. Circular saws can be provided with a guard of simple yet most efficient character, and emery wheels which "fly" sometimes, the scattering fragments carrying fearful destruction in their wake, are protected by safety plates. Women and girls who work in the tinware trades are also prevented, by small wire cages, from putting their hands below "the plunger" of the power press. Although the writer of the article referred to mentions all these means as being adopted to prevent injury, no indication is given of the extent to which they have been introduced into factories. Eighty thousand accidents a year seems a surprisingly great number. If that number were maintained annually for ten years the total would exceed the population of any town in England, with the exception of London. Many of such accidents are without doubt of comparatively slight character, yet not a few of those which do not result in permanent physical disablement occasion, especially in women, nervous shock. The results of such shock, a sequence of slight injury received in a factory, are sometimes seen in the out-patient department of a hospital. There may be functional nervous disease; or chorea, which so curiously in many instances is a sequence of fright, may be present; and chorea is unfortunately not always merely a passing morbid event; it too often leaves behind permanent disease of the heart. Our modern civilisation entails many ills. The introduction of machinery has certainly not been an unmixed blessing. But in our fight to keep our place among the nations of the world every modern invention must be utilised. In that fight, however, not only the instincts of humanity, but the colder reasoning prompted by economy, dictate the necessity of doing all that is possible to protect the life, limb, and health of the worker.

